# Inhibitors of the CD73-adenosinergic checkpoint as promising combinatory agents for conventional and advanced cancer immunotherapy

**DOI:** 10.3389/fimmu.2023.1212209

**Published:** 2023-06-26

**Authors:** Zoya Kurago, Gang Guo, Huidong Shi, Roni J. Bollag, Michael W. Groves, J. Kenneth Byrd, Yan Cui

**Affiliations:** ^1^ Department of Oral Biology and Diagnostic Sciences, Dental College of Georgia at Augusta University, Medical College of Georgia, Augusta University, Augusta, GA, United States; ^2^ Department of Biochemistry and Molecular Biology, Medical College of Georgia, Augusta University, Augusta, GA, United States; ^3^ Georgia Cancer Center, Medical College of Georgia, Augusta University, Augusta, GA, United States; ^4^ Department of Pathology, Medical College of Georgia, Augusta University, Augusta, GA, United States; ^5^ Department of Otolaryngology, Medical College of Georgia, Augusta University, Augusta, GA, United States

**Keywords:** CD73, NT5E, CD39, adenosine, A_2A_R, A_2B_R, immune checkpoint inhibitor, combination therapy

## Abstract

The cell surface enzyme CD73 is increasingly appreciated as a pivotal non-redundant immune checkpoint (IC) in addition to PD-1/PD-L1 and CTLA-4. CD73 produces extracellular adenosine (eADO), which not only inhibits antitumor T cell activity via the adenosine receptor (AR) A_2A_R, but also enhances the immune inhibitory function of cancer-associated fibroblasts and myeloid cells via A_2B_R. Preclinical studies show that inhibition of the CD73-adenosinergic pathway in experimental models of many solid tumors either as a monotherapy or, more effectively, in combination with PD-1/PD-L1 or CTLA-4 IC blockades, improves antitumor immunity and tumor control. Consequently, approximately 50 ongoing phase I/II clinical trials targeting the CD73-adenosinergic IC are currently listed on https://clinicaltrials.gov. Most of the listed trials employ CD73 inhibitors or anti-CD73 antibodies alone, in combination with A_2A_R antagonists, and/or with PD-1/PD-L1 blockade. Recent evidence suggests that the distribution of CD73, A_2A_R and A_2B_R in tumor microenvironments (TME) is heterogeneous, and this distribution affects CD73-adenosinergic IC function. The new insights have implications for the optimally effective, carefully tailored approaches to therapeutic targeting of this essential IC. In the mini-review, we briefly discuss the cellular and molecular mechanisms of CD73/eADO-mediated immunosuppression during tumor progression and therapy in the spatial context of the TME. We include preclinical data regarding therapeutic CD73-eADO blockade in tumor models as well as available clinical data from completed trials that targeted CD73-adenosinergic IC with or without PD-1/PD-L1 inhibitors and discuss factors that are potentially important for optimal therapeutic outcomes in cancer patients.

## 1 Introduction

CD73 is a type I transmembrane glycoprotein widely expressed on cell surfaces of smooth muscle, epithelium, endothelium, fibroblasts, neurons, and the immune system (1–3). Functionally, CD73 is a rate-limiting ecto-5’-nucleotidase (NT5E), which together with other cell surface ectonucleoside triphosphate diphosphohydrolases, such as CD39 (ENTPDase 1), dephosphorylate ATP released from stressed/damaged cells and produce extracellular adenosine (eADO) (1–3). CD73 plays a critical role in tissue homeostasis under physiological and pathological conditions, including epithelial and endothelial barrier function, neuronal function, as well as immunity and inflammation (4–6). The roles of CD73 in modulating tumorigenesis, angiogenesis, and metastasis are increasingly appreciated (7–9) such that it is now recognized as a critical cancer immune checkpoint (IC) non-redundant to PD-1/PD-L1 and CTLA-4 (10–14). Preclinical studies and early clinical trials reveal important breakthroughs as well as challenges. Here, we briefly describe the cellular and molecular events associated with the CD73-adenosinergic pathway, discuss the current status of therapeutic interventions that target the CD73-ADO axis, and propose potential ways to enhance cancer treatment outcomes.

## 2 The CD73-adenosinergic pathway in the tumor microenvironment

**Figure 1:**
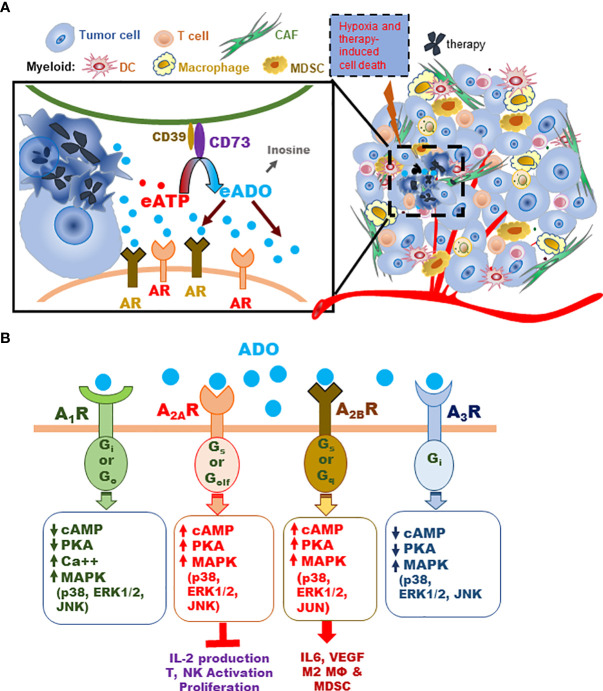
Schematic illustration of the CD73-adenosinergic pathway activity in the TME during tumor progression and treatment. **(A)** The CD39/CD73 enzyme pair converts ATP released by dying or stressed cells to immunosuppressive ADO, which inhibits antitumor immunity primarily by engaging A_2A_R and A_2B_R on various TME cells. **(B)** Schematic illustration of major AR signals activated by ADO. All four AR activate MAPK (p38, ERK1/2 and JNK or JUN) phosphorylation. Stimulatory A_1_R and A_3_R share several signaling events, including: A_1_R or A_3_R stimulation decreases adenylate cyclase activity and cAMP, inhibits protein kinase A (PKA), activates phospholipase C (PLCβ), and closes Ca^++^ channels. Stimulation of A_2A_R or A_2B_R produces opposite effects on adenylate cyclase, cAMP, and PKA (i.e. stimulates them) (4, 18, 19). Key functional impact of A_2A_R activity in TME effector T and NK cells are inhibition of activation, IL-2 production, and proliferation. The impact of ADO on A_2B_R-expressing myeloid and non-immune TME cells causes skewed pro-tumor phenotypes and activities that affect the TME and T/NK effector functions (4, 18, 19).

Hypoxia is a hallmark of the TME (15–17). Hypoxia and therapy-induced cell death potentiate ATP release into the extracellular space, which is rapidly metabolized by the CD39/CD73 enzyme-pair to ADO (**Figure 1A**). ADO acts on specific adenosine receptors (AR), A_1_R, A_2A_R, A_2B_R, and A_3_R. Stimulatory A_1_R and A_3_R are coupled with G_i_ or G_o_ proteins, whose activation suppresses cAMP with downstream immune-stimulatory effects. In contrast, A_2A_R and A_2B_R are coupled with G_s_ and/or G_olf_ or G_q_ proteins, which promote cAMP signaling and thus inhibit anti-tumor immune responses (**Figure 1B**) (4, 18, 19).

Preclinical and clinical studies show that in the TME, ADO mainly mediates immunosuppression via A_2A_R and A_2B_R due to hypoxia, inflammation and typically high ADO levels (4, 10, 13, 20). In particular, A_2A_R is highly expressed on T and NK cells and when activated, suppresses cell proliferation and effector function (13, 21–26). High A_2B_R levels on other TME cells potentiate immune suppressors including regulatory dendritic cells (DC), myeloid-derived suppressor cells (MDSC), tumor-associated macrophages (TAM) (13, 27–31), and cancer associated fibroblasts (CAF) (32). Moreover, A_2B_R augments CD73 expression on CAF via a CD73-A_2B_R-CD73 positive feedback loop, further exacerbating immunosuppression (13, 27–32).

## 3 CD73-adenosinergic pathway as a critical IC

### 3.1 Preclinical models

Preclinical studies targeting CD73 via genetic inactivation, neutralization, or small molecule inhibitors in numerous tumor models were reviewed extensively elsewhere (8–10, 13, 33). These studies reveal that the efficacy of anti-CD73 alone has limitations, in part because ADO can be generated by additional, though less prominent, pathways besides CD39/CD73. Similarly, clinical trials show that CD73 monotherapy is well-tolerated with moderate benefit in subsets of patients (9, 13, 34, 35). Treatment efficacy can be enhanced when CD73 targeting is combined with strategies to inhibit down-stream ADO signaling, among other methods. Here, we mainly focus on how these treatments impact specific AR-mediated cellular and molecular events that modulate the TME immune landscape.

#### 3.1.1 A_2A_R activity on T and NK cells

The critical inhibitory role of A_2A_R in T cell activation and antitumor immunity was first revealed by Ohta et al. in 2006 (22). This seminal study demonstrated that genetic inactivation of A_2A_R enhanced CD8 T cell-dependent antitumor immunity leading to the rejection of immunogenic tumors in ~60% of hosts without affecting the progression of non-immunogenic tumors (22). Subsequent studies revealed that ADO-induced A_2A_R signaling suppressed TCR-induced T cell activation including decreased production of IL-2, IFN-γ and TNF-α, which subsequently disrupted T cell proliferation and CD4 differentiation to Th1 and Th17 effectors (24, 36). Instead, A_2A_R activation promoted the generation of FoxP3^+^ and Lag-3^+^ regulatory T cells (Treg) and persistent T cell unresponsiveness to subsequent stimuli (24). A_2A_R signaling in CD8 T cells also interfered with Notch-1 upregulation and granzyme B production following TCR stimuli (23).

Early studies suggested that NK cell-dependent cytotoxicity was inhibited by CD73^+^ tumors leading to enhanced tumor metastasis (37, 38). Subsequent research revealed that CD73-A_2A_R activity suppressed NK cell maturation (39) and inhibited IL-15-induced NK cytotoxicity (40). Moreover, CD73^+^ NK cells within large tumors possessed immune-regulatory function via STAT3-induced IL-10, which suppressed CD4 T cell proliferation and IFN-γ production (41).

The immunosuppressive role of A_2A_R in T and NK cells was validated in preclinical murine tumor models. Genetic inactivation as well as A_2a_R antagonists alleviated T and NK cell unresponsiveness and enhanced antitumor immunity (37, 39, 42–45). A recent study employed the CRISPR/Cas9-mediated *A_2A_R* knockout in engineered human chimeric antigen receptor (CAR)-T cells, which made them resistant to ADO (46) and enhanced effector function and antitumor immunity *in vivo* in a preclinical model (46). These exciting results warrant clinical application of targeted A_2A_R inhibition/inactivation in T and NK cells to improve antitumor immunity and treatment outcomes.

#### 3.1.2 A_2B_R activity

Less is known about A_2B_R expression and function in various cell subsets and its relationship to CD73 in the TME. Unlike A_2A_R, A_2B_R has low affinity for ADO and is only activated by high ADO concentrations found under pathological conditions, which in the TME include hypoxia and therapy-induced cell stress or death (4, 5, 10, 47, 48). Notably, the expression of A_2B_R is markedly upregulated in response to hypoxia or inflammation (4, 20, 49). In the TME, A_2B_R is expressed in immune and non-immune cells, including myeloid cells, CAF, endothelium, and tumor cells (4, 18, 20, 32, 47). High levels of A_2B_R on some tumor cell types apparently promotes tumor proliferation, angiogenesis and metastasis (12, 47, 50, 51), which may be independent of immune regulation. In a glioblastoma model, a CD73-A_2B_R-CD73 positive feedback loop enhanced tumor chemoresistance (52).

So far, only myeloid cells, CAF and endothelium have been shown to exert ADO-A_2B_R-mediated immunosuppression. Moreover, a hypoxia-induced CD73-A_2B_R-CD73 positive feedback loop augmented CD73 and A_2B_R expression on endothelial cells (49). Much of this knowledge comes from *in vitro* studies and investigations of tissue damage in the absence of cancer (28, 48, 53), partly validated in the TME (27, 29, 47, 54). Activated A_2B_R in myeloid precursors promotes the immunosuppressive function of MDSC, differentiation of macrophages towards M2 phenotype, and induction of regulatory DC (27, 48, 54–57). These myeloid cells in turn inhibit antitumor T cell activity and promote angiogenesis and tumor metastasis potentially by reducing production of TNF-α and IL-12 while increasing IL-10, IL-6 and VEGF secretion (27, 58, 59).

Overall, these observations suggest an important immunosuppressive role for TME-associated A_2B_R and suggest that the CD73-A_2B_R-CD73 amplification loop may be a plausible therapeutic target.

#### 3.1.3 Combinatory targeting of A_2A_R and/or A_2B_R together with other IC

Preclinical evidence showed that while targeting individual A_2A_R- or A_2B_R-axis each positively impacted antitumor immunity and generally delayed tumor progression (60–62), we found that combined anti-CD73, A_2A_R- and A_2B_R-axis blockade markedly improved anti-tumor immunity with tumor regression (32). The specific reasons for the observed additive/synergistic effects are incompletely understood. Evidence does suggest that the effectiveness of anti-IC strategies depends upon the tumor type and TME conditions (13, 32). Furthermore, combinatory regimens that inhibit various CD73-AR axes and PD-1/PD-L1 are more effective, substantiating the non-redundant roles of CD73-AR IC and the advantage of targeting multiple IC (32, 61, 63).

### 3.2 Clinical observations

A large body of clinical evidence supports the negative impact of TME CD73 on cancer patient outcomes. Markedly elevated CD73 levels found in numerous tumors, including colorectal cancer (CRC), triple negative breast cancer (TNBC), head and neck cancer (HNC) and ovarian cancer (OC), have been linked to poor patient survival (64–70). Moreover, cancer therapies, including PD-1 ICB (71, 72), upregulated CD73-expression and ADO-AR signaling in the TME, potentially amplifying the role of this IC in patient outcomes (70, 73–76).

Several clinical studies have suggested that high blood levels of soluble (s)CD73, potentially generated by shedding, MMP-mediated clipping, or exome secretion, may prognosticate poor clinical outcome (77–79). However, a positive correlation between sCD73 and CD73 levels in the TME has not been established (77). While sCD73 has enzymatic activity in circulation, its’ impact on T cell-mediated antitumor immunity in the TME might be limited due to the spatial impact (addressed in the discussion). A mechanistic insight concerning sCD73 production and distribution in the TME is needed in order to fully understand its impact on antitumor immunity.

The specific roles of A_2A_R or A_2B_R and their relationship to CD73 expression in patient outcomes are less clear and possibly depend upon specific-tumor type, immunogenicity, and the TME landscape. On one hand, recent reports suggest that in non-small cell lung cancer (NSCLC), high A_2A_R expression in the TME independently predicted better patient overall survival (OS), while high CD73 levels were associated with poor OS (69). Similarly, high A_2A_R expression on CD8 T cells within OC nests correlated with durable clinical benefit/response (CBR) during a clinical trial of PD-1 ICB and an epigenetic modifier (80). On the other hand, the negative impact of A_2A_R on anti-tumor immunity was shown in a combined trial of PD-L1 and A_2A_R inhibitors in renal cell carcinoma (RCC), demonstrating better clinical responses to A_2A_R inhibitors when tumors exhibited high adenosine signature profiles (81). Additional studies are necessary to dissect the relationships of various CD73-AR axes and application to treatments.

## 4 Clinical trials targeting the CD73-adenosinergic pathway 

So far, approximately 50 active phase I/II cancer immunotherapy trials targeting the CD73-AR IC are listed on https://clinicaltrials.gov. Among these, more than 60% were designed to target CD73 by monoclonal antibodies or small molecule inhibitors, some of which were combined with PD-1/PD-L1 ICB regimens. The other 30-40% have employed small molecule inhibitors targeting A_2A_R alone or together with anti-CD73 and/or A_2B_R inhibitors (8, 61, 83–86). In addition to the safety (severity of adverse event, AE) and pharmacokinetic assessment of the therapeutic agents, a secondary objective was to collect data on clinical benefit rate (CBR), consisting of complete response (CR), partial response (PR) and stable disease (SD). Also, progression-free survival (PFS), objective response (OR), overall response rates (ORR) and overall survival (OS) were assessed based on standardized Response Evaluation Criteria in Solid Tumors version 1.1 (82) (for details, see legend to **Table 1**).

**Table 1:** List of registered clinical trials that target various aspects of CD73-adenosinergic axis.

anti-CD73 (mAb or small molecule inhibitor) +1- chemotherapy (w/anti-A2AR yellow)		anti-CD73 + anti-PDI/PDLI ICB +1- CTLA4 +1- A2aR inhibitor +/- chemotherapy (w/A2aR inhibitor – yeIlow)	
NCT03381274	Oleclumab (MED19447); AZD4635, Osimertinib	NCT02503774	Oleclumab MED19447 - MED14736
NCT03954704**	Dalutrafusp alfa; mFOLFOX6 Regimen	NCT02740985	Oleclumab, AZD4635; Durvalumab, Abiraterone Acetate; Enzalutamide; Docetaxel
NCT04797468	HLX23	NCT02754141	BMS-986179 - Nivolumab- rHuPH20
NCT05001347	TJ004309	NCT03267589	Oleclumab (MED19447); Durvalumab; Tremelilumab- MEDI 0562
NCT05143970	IPH5301; Trastuzumab	NCT03334617	Oleclumab (MED19447); Durvalumab
NCT05173792	AK119	NCT03454451	CPI-006; CPI-444; pembrolizumab
NCT05227144	ORIC-533	NCT03549000	NZV930 PDR00I- NIR178
A_2A_R antagonist		NCT03611556	Oleclumab (MED19d47); Durvalumab; Gemcitabine; Nab-paclitaxel
NCT02403193***	PBF-509; PDR00I	NCT03616886	Oleclumab (MED19447); MED14736; Paciitaxel; Carboplatin
NCT03207867***	NIR178, PDR00I	NCT03835949	TJ004309, Atezolizumab
NCT04895748***	MRI 78, PDR00I; DFF332; RADOOI	NCT03875573	Oleclumab (MED19447); Durvalumab; Radiatiom Stereotactic Body Radiotherapy
NCT05501054^$^	Ciforadenant; Nivolumab; Ipilimumab,	NCT04104672	AB680; Zimberelimab; Nab-paclitaxel; Gemcitabine
A_2B_R antagonist		NCT04148937	LY3475070 (CD73 inhibitor); Pembrolizumab
NCT03274479	PBF-1129	NCT04262388	Oleclumab MED19447; Durvalumab
NCT05234307****	PBF-1129; Nivolumab	NCT04381832*	Quemliclustat (AB680); Etrumadenant (AB928); Zimberelimab; Enzalutamide; Docetaxel; SG
NCT05272709	TT-702	NCT04572152	AK119; AK104
A_2A_R and A_2B_R antagonist		NCT04660812*	AB680; etrumadenant; zimberelimab, mFOLFOX-6 regimen; bevacizumab; regorafenib
NCT05024097***	Etrumadenant (AB928); Zimberelimab (AB122); Radiation therapy; FOLFOX	NCT04668300	Oleclumab (MED19447); Durvalumab
NCT05177770	Etrumadenant (AB928); Zimberelimab- SRF617	NCT04672434	Sym024; Sym021
NCT05198349	M1069	NCT04869501	TJ004309 Atezolizumab
A3R antagonist		NCT04989387	INCA00186; Retifanlimab; INCB106385
NCT00790218	CF-102 (CI-IB-MECA)	NCT05119998	IB1325; sintilimab
*w/A2AR/A2BR inhibitor; **dual anti-CD73/anti-TGF-beta; ***w/anti-PD1; ****w/anti-CD39/anti-PD1; ^$^w/anti-PD1/anti-CTLA4.		NCT05174585	JAB-BX102; mbrolizumab
		NCT05246995	IB1325; sintilimab
		NCT05329766	Quemliclustat; Zimberelimab; Domvanalimab; Fluorouracil; Leucovorin; Oxaliplatin
		NCT05431270	PT199; Q3W
		NCT05559541	AK119; AK104
		NCT05632328**	AGEN1423; Balstilimab; Gemcitabine; Nabpaclitaxel

Not listed in the table are >20 trials using non-selective inhibitors of multiple AR. Clinical trials record a variety of outcomes, according to standardized Response Evaluation Criteria in Solid Tumors (RECIST) version 1.1. Complete response (CR) = disappearance of all target lesions; partial response (PR) = at least a 30% decrease in the sum of diameters of target lesions; progressive disease (PD) = at least a 20% increase in the sum of diameters of target lesions, taking as reference the smallest sum on study; stable disease (SD) = neither sufficient shrinkage to qualify for PR nor sufficient increase to qualify for PD; objective response rates (OR) = overall response rates (ORR) = percentage of patients with partial or complete response; progression-free survival (PFS) = the length of survival without disease progression; overall survival (OS) = the length of survival time from either the date of diagnosis or the start of treatment. Adverse events (AE) are graded 1-5: Grade 1 - mild; asymptomatic or mild symptoms; Grade 2 - moderate; minimal, local or noninvasive intervention indicated; Grade 3 - severe or medically significant but not immediately life-threatening; Grade 4 - life-threatening consequences; urgent intervention indicated. Grade 5 - death related to AE.

### 4.1 Clinical trials targeting CD73

#### 4.1.1 Anti-CD73 antibodies

In approximately 20 trials in a variety of solid tumors, anti-CD73 monoclonal antibodies have been employed alone or more often, in combination with anti-PD-L1 or anti-PD-1. These antibodies are listed as Oleclumab, MEDI9447, AK119, HLX23, IPH5301, Sym042, CPI-0006, IBI325, PT199, JAB-BX102, TJ004309, NZV930, INCA00186 and BMS-986179. Most of the trials were/are phase I/Ib for safety assessment with limited preliminary reports of clinical outcomes in publications, abstracts, or oral presentations at international conferences, briefly described below.

In general, anti-CD73 caused low-grade AE classified as manageable or acceptable tolerability (NCT02503774, NCT03381274, NCT03616886, NCT03611556 and NCT03334617). Early reported outcomes have been mixed, as some showed promising signs of disease control, while others lacked solid evidence of clinical benefits. For instance, the NCT02503774 phase I trial of anti-CD73 with or without anti-PD-L1 enrolled 77 patients with CRC, 73 with pancreatic adenocarcinoma (PDAC) and 42 with NSCLC positive for EGFR mutation (EGFRm). Among those with evaluable outcomes, one CRC, two PDAC and four EGFRm NSCLC patients had OR, while nine CRC, eight PDAC and nine EGFRm NSCLC patients had SD. Overall, the antitumor activity was promising in EGFRm NSCLC patients receiving anti-CD73/anti-PD-L1 therapy, whereas the effectiveness in CRC and PDAC is yet to be verified (34).

The NCT03381274 phase Ib/II study evaluated the effects of anti-CD73 combined with third-generation tyrosine kinase inhibitors (TKI) in advanced EGFRm NSCLC in previously treated patients and reported acceptable tolerability. Clinical observations up to July 2021 were published in 2023 for patients with T790M-negative EGFRm NSCLC and showed CBR of 75% and OR of 25% in five patients receiving 1500 mg anti-CD73 antibody; CBR of 82.4% and OR of 11.8% were noted in 21 patients administered 3000 mg anti-CD73 antibody (35). For patients on the higher dose of anti-CD73, the median PFS was 7.4 months as compared with PFS of 2.8 months without anti-CD73 (35).

Similar studies include NCT03616886 phase I/II trial testing anti-CD73, anti-PD-L1 and chemotherapy in subjects with advanced TNBC (87), NCT03611556 phase Ib/II trial testing anti-CD73 alone or combination with gemcitabine chemotherapy and anti-PD-L1 in 212 patients with metastatic PDAC, and NCT03334617 HUDSON Platform multi-arm phase II trial for NSCLC patients who previously failed anti-PD(L)1 immunotherapy. These trials have yet to report the results (NCT03611556), or else had limited patient numbers (87) (NCT03616886) or short treatment duration (NCT03334617) (88) insufficient to assess clinical benefits.

The NCT03954704 phase I trial initiated in 2019 differs from others by testing a bi-functional antibody against CD73 and TGFβ (known as GS-1423 and AGEN1423) in patients with advanced solid tumors (89). Because TGF-β is an immunosuppressive cytokine that enhances ADO-mediated CD73 upregulation (76, 90–92), it is expected to improve treatment efficacy. Early assessment in 21 patients showed AE ranging from mild to severe, including death (89). In patients administered a high dose (20-45 mg/Kg), the circulating bi-functional antibody was durable and effectively bound to B cell CD73. Among the 17 patients who reached the first response assessment, 4.8% had a PR, 33.3% had SD, and 42.9% showed progressive disease (PD) (89).

#### 4.1.2 CD73 small molecule inhibitors

Besides anti-CD73 antibody, small molecule inhibitors specific for CD73, AB680, ORIC-533 and LY3475070, have been employed. The NCT04104672 phase I/Ib trial was designed to evaluate safety and tolerability of AB680 with chemotherapy (paclitaxel and gemcitabine) and anti-PD-1 for treatment-naive patients with metastatic mPDAC (93). Preliminary observations in 13 patients receiving various doses of AB680 showed a manageable safety profile with AE up to grades 3-4. Early clinical responses among nine evaluable patients included three PR and five SD (93).

### 4.2 Trials targeting A_2A_R or A_2B_R

The A_2A_R antagonists AZD4635, NIR178 and Ciforadenant (CPI-444) were developed to block the A_2A_R-mediated inhibition of T and NK activity. The A_2B_R antagonists PBF-1129 and TT-702 as well as the dual A_2A_R/A_2B_R antagonists Etrumadenant (AB928) and M1069 were developed to target the myeloid, stromal and potential tumor cell-mediated immunosuppression for additive/synergistic effects of dual CD73-AR axes blockade.

Fong et al. reported the results of A_2A_R inhibitor CPI-444 phase I trial NCT03454451 in 68 patients with RCC (81), 33 of which received CPI-444 alone and 35 received both CPI-444 and anti-PD-L1. *In both groups*, the regimens were safe and improved overall survival with durable clinical benefit associated with increased CD8^+^ T cell recruitment into the tumors and broadened circulating T-cell repertoires (81). Remarkably, better clinical response was associated with enriched adenosine-related gene-expression profile in pre-treatment RCC specimens (81), supporting the hypothesis that elevated CD73-AR signaling is a targetable non-redundant IC, and its blockade may enhance antitumor immunity. Moreover, adenosine-regulated gene signature may be a useful marker to predict clinical prognosis (69, 80).

Early results of the NCT02740985 phase Ia/b trials using A_2A_R inhibitor AZD4635 alone or in combination with anti-PD-L1 antibody in 250 PD-1/PD-L1 inhibitor-naive patients with advanced solid tumors, including metastatic castration-resistant prostate cancer (mCRPC), CRC or NSCLC were reported recently (94). Both monotherapy and combination therapy were well tolerated with an overall <20% above grade 3 AE. ORR was observed in ~5% of the 39 mCRPC patients on AZD4635 monotherapy and ~16.2% of 37 patients on combination therapy (94). This trial also revealed a positive correlation between high adenosine signature in the blood and better clinical response, as 24-week PFS was noted in 48.9% of high adenosine-signature patients versus 20.8% of low adenosine-signature patients (94).

Clinical trials that target the A_2B_R are limited. The NCT04381832 phase Ib/II trial to evaluate the A_2A_R/A_2B_R dual antagonist AB928 with or without anti-PD-1 and chemotherapy in patients with mCRPC reported a manageable safety profile in 17 enrolled patients (95). Among 16 patients that continued with AB928 treatment, the composite ORR was 43% (95).

## 5 Discussion and future perspectives

### 5.1 Combinatory regimens of CD73-ICB with other therapies

The early clinical observations of CD73-AR ICB trials have demonstrated feasibility, manageable toxicity and promising potential for tumor control. The benefits of anti-CD73 monotherapy appear modest, but markedly improved when combined with PD-1/PD-L1 ICB and/or other cancer therapies. As CD73-IC is continuously activated and exacerbated by hypoxia and therapy-induced cell death (32, 80, 81, 94), targeting multiple CD73-AR axes in the context of conventional or advanced therapies will improve therapeutic benefits. In particular, CD73-ICB before and during cell death induced by therapy will promote eATP-mediated antitumor immunity (96, 97). While current CD73-AR ICB trials include chemotherapy-treated patients, future trials designed to target CD73-IC aspects specific to the patient and the TME may significantly improve outcomes.

### 5.2 Targeting strategies based on spatial context of CD73-AR axes

Productive antitumor immunity relies on direct interactions between effector and tumor cells. Recent studies have illustrated that close effector-target cell proximity in the TME directly affects clinical outcomes (98, 99). As eADO stability and diffusion are limited, the expression levels, distribution and proximity of CD73, A2AR and A2BR in the TME will determine the activity of specific CD73-AR axes and the mechanisms of ADO-mediated immunosuppression. For example, in the TME with few T and NK cells, CD73-A2AR axis might be insignificant despite high prevalence of CD73. Yet, in the absence of the spatial distribution mapping, it is unclear which aspects of ADO-mediated immunosuppression, and at what stage of treatment, would be most relevant. We propose that spatial distribution maps of these receptors, combined with the knowledge of relevant cellular compartments, could be important tools to identify key pathways of ADO-mediated immunosuppression operating in various TME over time and inform the design of CD73-IC targeting strategies.

In conclusion, tremendous advances have occurred in the area of CD73-AR ICB in the past decade. Combined ICB strategies targeting CD73-AR and PD-1/PD-L1 with conventional or advanced therapies remain a promising and exciting area of research. Further advances will be made possible through better understanding of the tumor-specific and treatment-specific TME, including the spatial distribution of CD73, A_2A_R and A_2B_R.

## Author contributions

ZK, GG and YC reviewed the literature and wrote the manuscript. HS, RB, MG, and KB participated in the discussion and revision. All authors contributed to the article and approved the submitted version.
